# Data on changes in red wine phenolic compounds and headspace aroma compounds after treatment of red wines with chitosans with different structures

**DOI:** 10.1016/j.dib.2018.02.029

**Published:** 2018-02-16

**Authors:** Luís Filipe-Ribeiro, Fernanda Cosme, Fernando M. Nunes

**Affiliations:** a CQ-VR, Chemistry Research Centre, Food and Wine Chemistry Lab, University of Trás-os-Montes and Alto Douro, School of Life Sciences and Environment, Chemistry Department, 5000-801 Vila Real, Portugal; b CQ-VR, Chemistry Research Centre, Food and Wine Chemistry Lab, University of Trás-os-Montes and Alto Douro, School of Life Sciences and Environment, Biology and Environment Department, 5000-801 Vila Real, Portugal

**Keywords:** Red wine, 4-Ethylphenol, 4-Ethylguaiacol, Chitosan, Chitin, Chromatic characteristics, Phenolic compounds, Headspace aroma abundance

## Abstract

Data in this article presents the changes on phenolic compounds and headspace aroma abundance of a red wine spiked with 4-ethylphenol and 4-ethylguaiacol and treated with a commercial crustacean chitin (CHTN), two commercial crustacean chitosans (CHTB, CHTD), one fungal chitosan (CHTF), one additional chitin (CHTNA) and one additional chitosan (CHTC) produced by alkaline deacetylation of CHTN and CHTB, respectively. Chitin and chitosans presented different structural features, namely deacetylation degree (DD), average molecular weight (MW), sugar and mineral composition (“Reducing the negative sensory impact of volatile phenols in red wine with different chitosan: effect of structure on efficiency” (Filipe-Ribeiro et al., 2018) [1]. Statistical data is also shown, which correlates the changes in headspace aroma abundance of red wines with the chitosans structural features at 10 g/h L application dose.

**Specifications Table**

**Table:** 

Subject area	*Chemistry*
More specific subject area	*Food and Wine Chemistry*
Type of data	*Table, graph, figure*
How data was acquired	*X-ray (PANalytical X’Pert Pro X-ray diffractometer equipped with X’Celerator detector and secondary monochromator)*
	*FTIR (Unicam Research Series)*
	*HPLC (Ultimate 3000, Dionex) with a Photodiode array detector (PDA-100, Dionex)*
	*GC–MS (Thermo-Finningam) with CombiPAL automated HS-SPME (CTCANALYTICS, AG)*
	*HPAEC-PAD (ICS-3000, Dionex)*
Data format	*Analysed*
Experimental factors	*Wine sample was spiked with two levels of 4-ethylphenol (750* *μg/L and 1500* *μg/L) and 4-ethylguaicol (150* *μg/L and 300* *μg/L) and treated with chitosan with different characteristics and application doses (10, 100 and 500* *g/h L).*
Experimental features	*Chitin and chitosan were analysed by titration, viscosimetry, sugar analysis, X-Ray diffraction and FTIR for their characterization*
	*Wine phenolic acids and anthocyanins were analysed by RP-HPLC with a photodiode array detector and headspace aroma abundance were analysed by headspace solid phase microextraction using a 50/30* *μm DVB/Carboxen/PDMS fibre followed by GC–MS using an Optima FFAP column (30 m×0.32* *mm, 0.25* *μm).*
Data source location	*Vila Real, Portugal*
Data accessibility	*Data with this article*

**Value of the data**

● Data presented in this study shows the effect of chitins and chitosans physicochemical characteristics on the phenolic composition, headspace aroma abundance of wines spiked with 4-ethylphenol and 4-ethylguaiacol.
● Red wines treated with chitins and chitosans with distinct physicochemical characteristics and application doses (10, 100 and 500 g/h L) were analysed by RP-HPLC to determine the phenolic profile and by HS-SPME-GC/MS to analyse the aroma compounds.
● Chitins and chitosans reduced the headspace abundance of 4-ethylphenol and 4-ethylguaiacol of red wine, and the reduction was dependent on the deacetylation degree of chitins and chitosans and on their source (fungal *vs* crustacean origin).
● Increased application doses decreased headspace aroma abundance and phenolic compounds.
● This data could serve as a benchmark for other researchers, evidencing the influence of chitins and chitosans treatment and dose applied on the individual phenolic compounds, chromatic characteristics and headspace aroma abundance of red wine.

## 1 Data

**Fig. 1:**
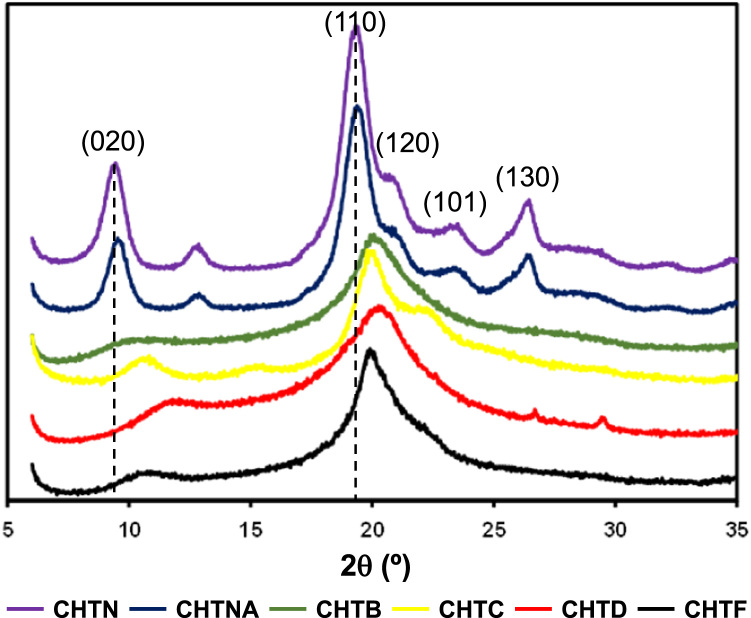
X-ray diffraction patterns of chitins and chitosans.

**Fig. 2:**
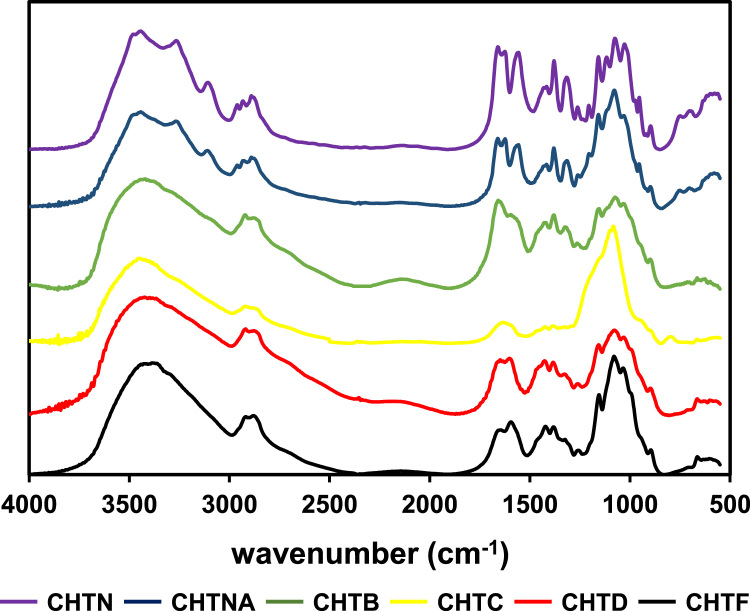
FTIR spectra of chitins and chitosans.

**Fig. 3:**
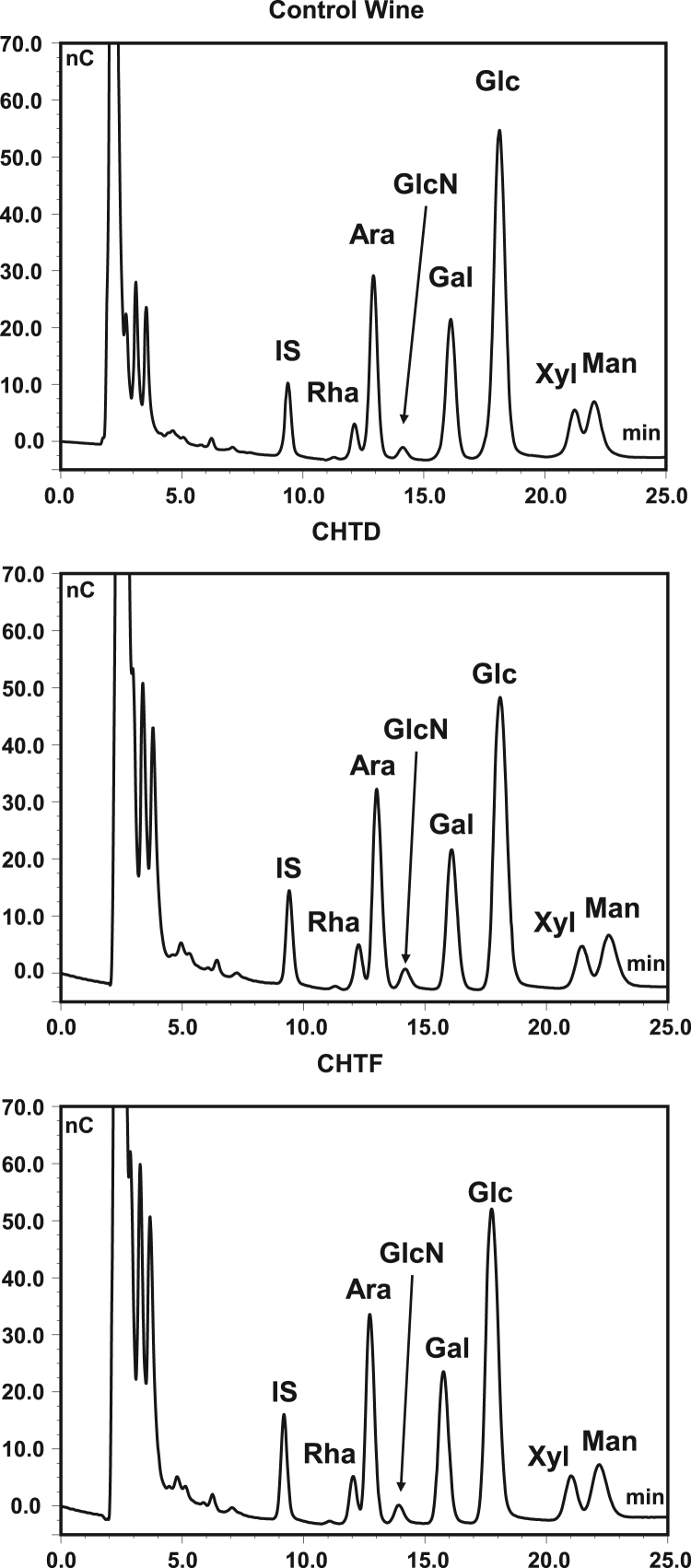
Chromatograms obtained by acid hydrolysis of wines before and after application of 10 g/L of chitosan CHTD (crustacean origin) and CHTF (fungal origin). IS-internal standard (2-deoxyglucose); Rha – rhamnose; Ara – arabinose; GlcN – glucosamine; Gal – galactose; Glc – glucose; Xyl – xylose; Man – mannose.

The data reported includes information about X-Ray diffraction pattern of chitins and chitosans (Fig. 1), FTIR spectra (Fig. 2) and band assignments of chitins and chitosans (Table 1), amount of chitosan dissolved in red wine when applied at 10, 100 and 500 g/h L (Fig. 3 and Table 2). The headspace aroma abundance of red wines before and after treatment at 10, 100 and 500 g/h L application doses of crustacean (CHTD) and fungal (CHTF) chitosans were determined (Table 3) and the correlation between the headspace aroma compounds abundance reduction with the chitins and chitosans deacetylation degree was calculated (Table 4). Total phenols, flavonoid phenols, non-flavonoid phenols, total anthocyanins, colour intensity, hue and chromatic characteristics of treated and untreated wines were determined (Table 5). Phenolic acids and flavonoids of wines were determined by RP-HPLC (Table 6) and monomeric anthocyanins (Table 7) for 10 g/h L application doses. Total phenols, flavonoid phenols, non-flavonoid phenols, total anthocyanins, colour intensity, hue and chromatic characteristics for red wines before and after treatment with 10, 100 and 500 g/h L application doses of crustacean (CHTD10, CHTD100 and CHTD500, respectively) and fungal (CHTF10, CHTF100 and CHTF500, respectively) chitosans were determined (Table 8). Phenolic acids and flavonoids (Table 9) and monomeric anthocyanins (Table 10) of wines before and after treatment with 10, 100 and 500 g/h L application doses of crustacean (CHTD) and fungal (CHTF) chitosans were determined by RP-HPLC.

**Table 1:** Characteristic absorption bands (FTIR) and their assignment in chitins and chitosans used with different physicochemical characteristics.

CHTN	CHTNA	CHTB	CHTC	CHTD	CHTF	Assignment [2], [3]
3487	3485					υOH
3452	3458	3454	3465	3440	3433	υOH
3275	3275					υasNH
3114	3122					υsNH
2964	2964					υasCH3
2937	2939	2923	2937	2929	2926	υasCH2
2893	2894	2891	2861	2896	2891	υCH3
1658	1658	1662	1655	1660	1662	υC=O (Amide I)
1624	1624					υC=O (Amide I)
1560	1565	1612	1601	1612	1599	υC-N (C-N-H)+δNH (Amide II)
1435	1440	1433	1433	1435	1427	δCH2
1431	1427					
1381	1381	1385	1396	1389	1389	δCH+ δC-CH3
1317	1321	1329	1340	1336	1335	υC-N +δNH (Amide III)
1263	1263	1286	1295	1269	1265	δNH
1207	1205					
1157	1157	1157	1147	1159	1157	υsC-O-C (glycosidic linkage)
1119			1104			υC-O
1078	1083	1082	1084	1084	1082	υasC-O-C (glycosidic linkage)
1030	1041	1039			1037	υC-O
982	972					γCH3
955			945			
899	902	898	900	902	901	γCH (C1-axial) (β-bond)

**Table 2:** Amount of glucosamine$ (g/h L) in red wines before and after treatment with chitosans (CHTD and CHTF) with different physicochemical characteristics and application doses.

	Glucosamine (g/h L)	Chitosan dissolved (g/h L)	Percentage of dissolved chitosan
TF	1.36±0.10^a^		
			
CHTD			
10 g/h L	2.13±0.22^b,c^	0.77	7.70%
100 g/h L	2.46±0.09^c,d^	1.10	1.10%
500 g/h L	2.68±0.16^d^	1.32	0.26%
			
CHTF			
10 g/h L	1.99±0.27^b^	0.63	6.3%
100 g/h L	2.23±0.25^b,c^	0.87	0.87%
500 g/h L	2.35±0.09^b,c,d^	0.99	0.20%

$ Expressed as anhydrosugar; Means within a column followed by the same letter are not significantly different ANOVA and Tukey post-hoc test (*p*<0.05).

**Table 3:** Headspace aroma abundance of red wines (volatile phenols free T0 and volatile phenols spiked with 750 µg/L of 4-EP and 150 µg/L of 4-EG, TF) after treatment with chitosans with different physicochemical characteristics and application doses.

**Compounds**	**ID**	**RI Calculated**	**RI**	**MW (g/mol)**	**Odour descriptor**	**ODT (mg/L)**	**T0**	**TF**	**CHTD10**	**CHTD100**	**CHTD500**	**CHTF10**	**CHTF100**	**CHTF500**
Ethyl acetate	…	710	715	88.11	Fruity, sweet	7.5	850.94±23.71^a^	768.10±23.98^ab^	716.87±57.29^bc^	717.26±16.15^bd^	430.35±16.30^e^	768.43±62.57^acd^	758.26±32.68^cd^	492.87±30.72^e^
2-Methylpropan-1-ol	…	1094	1114	74.12	Bitter,green, harsh	0.2	249.26±30.87^a^	216.29±10.36^ab^	184.38±18.61^cde^	164.50±9.73^cf^	142.45±5.70^dg^	211.76±5.00^bf^	168.40±14.41^efg^	158.29±10.50^efg^
3-Methylbutan-1-ol-acetate	Std	1176	1126	130.18	Banana	0.03	5.15±0.21^a^	4.72±0.53^a^	2.41±1.17^bc^	1.76±0.18^bd^	n.d.	4.16±0.21^ae^	3.04±0.41^ce^	2.81±0.50^cd^
Ethyl octanoate	Std	1410	1429	172.27	Sweet, fruity, fresh	0.005	98.19±4.10^a^	92.62±1.37^a^	30.99±2.77^b^	25.97±2.24^b^	17.78±2.06^c^	73.19±4.03^e^	40.30±4.30^d^	25.54±2.24^b^
Ethyl decanoate	Std	1594	1630	200.32	Grape	0.2	35.47±11.20^a^	32.18±9.05^a^	n.d.	n.d.	n.d	14.46±1.69^b^	6.59±1.01^b^	6.10±2.05^b^
Diethyl succinate	Std	1650	1698	174.19	Light fruity	7.5	241.51±22.06^a^	231.43±15.30^a^	131.63±19.76^bc^	128.44±5.01^b^	118.66±3.70^b^	193.29±18.65^d^	169.06±17.15^d^	166.01±7.02^cd^
2-Phenylethanol	Std	1920	1911	122.16	Roses, sweet	14.0	634.30±79.82^a^	553.13±16.48^ab^	364.27±31.95^cd^	355.88±35.74^ce^	336.02±32.43^cg^	485.25±9.23^bf^	425.70±32.97^def^	397.35±18.50^efg^
4-Ethylguaiacol	Std	1870	1989	152.18	Smoke	0.15	…	3.62±0.18^a^	2.52±0.23^bc^	2.35±0.14^bd^	2.18±0.27^be^	3.24±0.14^af^	2.79±0.09^cdef^	2.64±0.35^ef^
*Reduction (%) SPME*							…	…	*30.4*±*2.77*^*ab*^	*35.0*±*2.08*^*bc*^	*38.9*±*2.25*^*c*^	*10.5*±*0.45*^*d*^	*22.9*±*0.74*^*a*^	*27.2*±*1.87*^*a*^
Octanoic acid	Std	2040	2030	144.21	Fatty acid, rancid	0.5	22.90±16.28^a^	16.28±0.63^b^	9.02±0.33^cdf^	8.01±0.92^ce^	7.30±0.56^ce^	12.60±1.66^g^	10.52±0.77^dfg^	10.34±1.42^dfg^
4-Ethylphenol	Std	2100	2142	122.16	Musty, spicy, phenolic	0.4	…	10.97±0.48^a^	7.82±0.43^bc^	7.00±0.58^bde^	6.67±0.12^bdf^	9.28±0.37^g^	7.92±0.34^cef^	7.67±0.77^cg^
*Reduction (%) SPME*							…	…	*28.7*±*1.58*^*a*^	*36.2*±*2.99*^*bc*^	*39.2*±*0.55*^*c*^	*15.4*±*0.61*^*d*^	*27.8*±*1.19*^*a*^	*30.1*±*1.81*^*ab*^
Decanoic acid	Std	2170	2196	172.27	Fatty, rancid, soap	1.0	12.97±0.65^a^	10.86±3.36^b^	5.44±1.33^cd^	4.20±0.04^cef^	3.93±0.41^cgh^	6.10±0.51^dg^	4.56±0.02^deg^	2.20±0.42^efh^
**Total area – VPs area**							2150.69±25.30	1940.18±8.23^a^	1455.35±17.76^b^	1415.37±10.44^b^	1143.44±9.99^c^	1807.66±17.19^d^	1647.84±12.29^e^	1329.12±9.10^f^
***Reduction (%) SPME***							…	….	**22.5±0.26**	**22.6±0.16**	**41.06±0.37**	**6.8±0.06**	**15.1±0.11**	**31.5±0.22**

Results expressed in absolute area (area*10^5^). Values are presented as mean±standard deviation; $ ID – Identification; Std – Standard; * RI (retention index) from: Vás et al. [4]; Bailley et al. [5]; Czerny et al. [6]. MW (molecular weight). ODT (olfactory detection threshold) and odour descriptor from: Perestrelo et al. [7]; Dragone et al. [8]. Jiang and Zhang [9]. Means within a column followed by the same letter are not significantly different ANOVA and Tukey post-hoc test (*p*<0.05). n.d., not detected; Uncontaminated (T0) spiked red wine (TF) and wines treated with chitosans. VPs – volatile phenols. Crustacean chitosan CHTD10 (10 g/h L), CHTD100 (100 g/h L), CHTD500 (500 g/h L) and fungal chitosan CHTF10 (10 g/h L), CHTF100 (100 g/h L) and CHTF500 (500 g/h L).

**Table 4:** Correlations between headspace abundance of wine aroma compounds and deacetylation degree of chitins and chitosans applied at 10 g/h L.

	Pearson Correlations	Spearman Correlations	Gamma Correlations	Kendall Tau Correlations
Ethyl acetate	−0,925*	−1,00*	−1,00*	−1,00*
3-Methylbutan-1-ol acetate	−0,790	−1,00*	−1,00*	−1,00*
2-Methyl-1-butan-1-ol	0,041	0,000	−0,200	−0,200
Ethyl hexanoate	−0,546	−0,400	−0,400	−0,400
1-Hexanol	−0,981*	−0,975*	−1,00*	−0,949*
Ethyl octanoate	−0,754	−0,900*	−0,800*	−0,800*
Ethyl decanoate	−0,659	−0,800	−0,600	−0,600
Diethyl succinate	−0,986*	−1,00*	−1,00*	−1,00*
Phenylethyl acetate	−0,985*	−1,00*	−1,00*	−1,00*
Ethyl dodecanoate	−0,509	−0,500	−0,400	−0,400
Hexanoic acid	−0,874	−0,900*	−0,800*	−0,800*
Benzyl alcohol	−0,960*	−1,00*	−1,00*	−1,00*
2-Phenylethanol	−0,975*	−0,900*	−0,800*	−0,800*
4-Ethylguaiacol (4-EG)	−0,974*	−0,900*	−0,800*	−0,800*
Octanoic acid	−0,871	−0,800	−0,600	−0,600
4-Ethylphenol (4-EP)	−0,989*	−1,00*	−1,00*	−1,00*
Decanoic acid	−0,719	−0,700	−0,600	−0,600
Dodecanoic acid	−0,974*	−1,00*	−1,00*	−1,00*

* *p*<0.05.

**Table 5:** Total phenols, flavonoid phenols, non-flavonoid phenols, total anthocyanins and chromatic characteristics of red wines before (TF) and after treatment with chitins and chitosans with different physicochemical characteristics.

**Samples**	**Total phenols**	**Flavonoid phenols**	**Non-flavonoid phenols**	**Total anthocyanins**	**Colour intensity**	**Hue**	L*	a*	b*	C*	**°*h***	**Δ*E***
	**(mg/L gallic acid)**	**(mg/L gallic acid)**	**(mg/L gallic acid)**	**(mg/L)**	**A.U.**							
**TF**	1907±49^a^	1534±58^a^	373±9^a^	343±0^a^	12.27±0.25^a^	0.66±0.01^a^	6.41±0.81^a^	34.53±1.72^a^	32.41±1.53^a^	47.36±2.30^a^	0.75±0.00^a^	…
**CHTN10**	1963±73^a^	1598±61^a^	365±12^a^	342±7^a^	12.09±0.94^a^	0.66±0.01^a^	7.17±2.43^a^	35.60±5.01^a^	33.34±4.25^a^	48.77±6.56^a^	0.75±0.01^a^	1.60±2.26^a^
**CHTNA10**	1936±58^a^	1574±61^a^	362±4^a^	349±6^a^	11.71±0.07^a^	0.66±0.00^a^	8.47±0.27^a^	37.50±0.58^a^	35.01±0.52^a^	51.30±0.78^a^	0.75±0.00^a^	4.44±0.83^a^
**CHTB10**	1851±4^a^	1509±29^a^	342±33^a^	343±1^a^	11.18±0.08^a^	0.65±0.00^a^	10.59±0.31^a^	40.57±0.63^a^	37.56±0.59^a^	55.28±0.86^a^	0.75±0.00^a^	8.96±3.54^a^
**CHTC10**	1936±19^a^	1547±7^a^	388±12^a^	345±4^a^	12.62±0.56^a^	0.68±0.01^a^	5.30±1.18^a^	32.83±2.47^a^	31.27±1.92^a^	45.34±3.11^a^	0.76±0.01^a^	2.33±1.00^a^
**CHTD10**	1898±81^a^	1528±34^a^	370±47^a^	347±7^a^	12.09±0.63^a^	0.67±0.01^a^	7.23±1.58^a^	35.71±3.29^a^	33.63±2.68^a^	49.05±4.23^a^	0.76±0.01^a^	1.88±2.15^a^
**CHTF10**	1831±16^a^	1440±19^a^	387±10^a^	351±1^a^	12.16±0.30^a^	0.69±0.02^a^	7.22±1.52^a^	34.41±0.05^a^	32.32±0.70^a^	48.98±2.96^a^	0.75±0.01^a^	0.82±0.38^a^

Values are presented as mean±standard deviation; Means within a column followed by the same letter are not significantly different (Tukey, *p*<0.05). L* – lightness, a* - redness, b* - yellowness, ΔE* –colour difference. The values corresponding to ΔE* were obtained taking as a reference the untreated wine (TF). A.U. – absorbance units, spiked red wines (TF) and wines treated with chitins (CHTN, CHTNA at 10 g/h L) and chitosans (CHTB, CHTC, CHTD, CHTF at 10 g/h L).

**Table 6:** Phenolic acids and flavonoids of red wines spiked with volatile phenols (TF) and after treatment with chitins and chitosans with different physicochemical characteristics.

**Samples**	**Gallic acid**	**Catechin**	***trans*****-caftaric acid**	**GRP**	**Coutaric acid**	**Caffeic acid**	***p*****-Coumaric acid**	**Ferulic acid**	**Caffeic acid ethyl ester**	***p*-Coumaric acid ethyl ester**
	**(mg/L)**	**(mg/L)**	**(mg/L)**	**(mg/L)**	**(mg/L)**	**(mg/L)**	**(mg/L)**	**(mg/L)**	**(mg/L)**	**(mg/L)**
**TF**	31.87±0.10^a^	17.17±0.05^b^	30.81±0.05^c^	n.d.	12.34±0.02^a^	4.27±0.01^a^	1.72±0.03^a^	2.48±0.01^a^	0.27±0.00^a^	3.43±0.02^a^
**CHTN10**	30.08±0.30^a^	17.00±0.29^ab^	30.64±0.37^bc^	n.d.	12.25±0.13^a^	4.27±0.07^a^	1.70±0.04^a^	2.46±0.05^a^	0.27±0.03^a^	3.33±0.01^a^
**CHTNA10**	31.22±0.17^a^	16.76±0.00^ab^	30.10±0.17^ab^	n.d.	12.18±0.09^ab^	4.41±0.22^a^	1.68±0.03^a^	2.45±0.01^a^	0.26±0.03^a^	3.35±0.02^a^
**CHTB10**	29.81±1.91^a^	16.85±0.06^ab^	30.00±0.03^ab^	n.d.	12.12±0.04^ab^	4.26±0.00^a^	1.64±0.03^a^	2.51±0.07^a^	0.28±0.01^a^	3.35±0.04^a^
**CHTC10**	31.10±3.14^a^	16.69±0.03^a^	30.08±0.01^ab^	n.d.	12.20±0.05^ab^	4.41±0.20^a^	1.92±0.43^a^	2.49±0.09^a^	0.29±0.01^a^	3.37±0.02^a^
**CHTD10**	28.12±0.68^a^	16.56±0.03^a^	29.65±0.17^a^	n.d.	12.10±0.05^ab^	4.18±0.00^a^	1.61±0.02^a^	2.44±0.01^a^	0.27±0.03^a^	3.32±0.06^a^
**CHTF10**	31.33±0.31^a^	16.77±0.08^ab^	30.22±0.11^abc^	n.d.	11.96±0.05^b^	4.35±0.03^a^	1.89±0.04^a^	2.52±0.02^a^	0.29±0.01^a^	3.34±0.02^a^

Values are presented as mean ± standard deviation; Means within a column followed by the same letter are not significantly different (Tukey, *p*<0.05).

GRP - 2-*S*-glutathionyl caftaric acid. Spiked red wine (TF) and wine treated with chitins (CHTN, CHTNA at 10 g/h L) and chitosans (CHTB, CHTC, CHTD, CHTF at 10 g/h L).

**Table 7:** Monomeric anthocyanin composition of spiked red wines (TF) and after treatment with chitins and chitosans with different physicochemical characteristics.

**Samples**	**Del-3-Glc**	**Cya-3-Glc**	**Pet-3-Glc**	**Peo-3-Glc**	**Mal-3-Glc**	**Del-3-AcGlc**	**Cya-3-AcGlc**	**Pet-3-AcGlc**	**Peo-3-AcGlc**	**Mal-3-AcGlc**	**Del-3-CoGlc**	**Cya-3-CoGlc**	**Pet-3-CoGlc**	**Peo-3-CoGlc**	**Mal-3-CoGlc**
	**(mg/L)**	**(mg/L)**	**(mg/L)**	**(mg/L)**	**(mg/L)**	**(mg/L)**	**(mg/L)**	**(mg/L)**	**(mg/L)**	**(mg/L)**	**(mg/L)**	**(mg/L)**	**(mg/L)**	**(mg/L)**	**(mg/L)**
**TF**	0.54±0.02^a^	7.46±0.18^a^	10.04±0.08^a^	4.40±0.05^a^	78.53±1.79^a^	1.81±0.02^a^	n.d.	n.d.	0.65±0.02^a^	11.65±1.25^c^	n.d.	n.d.	n.d.	0.71±0.03^a^	12.13±0.12^a^
**CHTN10**	0.53±0.01^a^	7.82±1.35^a^	10.24±0.42^a^	4.35±0.10^a^	78.11±0.80^a^	1.77±0.06^a^	n.d.	n.d.	0.62±0.05^a^	10.82±0.69^bc^	n.d.	n.d.	n.d.	0.73±0.05^a^	11.86±0.43^a^
**CHTNA10**	0.53±0.01^a^	7.39±0.03^a^	9.89±0.04^a^	4.32±0.03^a^	78.30±0.24^a^	1.77±0.02^a^	n.d.	n.d.	0.61±0.02^a^	10.57±0.29^abc^	n.d.	n.d.	n.d.	0.72±0.02^a^	11.85±0.13^a^
**CHTB10**	0.54±0.01^a^	7.38±0.05^a^	9.92±0.03^a^	4.36±0.02^a^	77.96±0.48^a^	1.76±0.01^a^	n.d.	n.d.	0.61±0,00^a^	9.53±0.02^ab^	n.d.	n.d.	n.d.	0.70±0.01^a^	11.79±0.13^a^
**CHTC10**	0.54±0.01^a^	7.05±0.77^a^	9.67±0.59^a^	4.32±0.02^a^	78.12±0.23^a^	1.77±0.02^a^	n.d.	n.d.	0.61±0.02^a^	9.45±0.09^a^	n.d.	n.d.	n.d.	0.71±0.01^a^	11.81±0.00^a^
**CHTD10**	0.54±0.01^a^	7.14±0.37^a^	10.02±0.07^a^	4.33±0.03^a^	77.90±1.98^a^	1.75±0.03^a^	n.d.	n.d.	0.81±0.01^a^	9.45±0.25^a^	n.d.	n.d.	n.d.	0.70±0.07^a^	11.67±0.31^a^
**CHTF10**	0.55±0.01^a^	6.54±0.57^a^	9.70±0.23^a^	4.30±0.08^a^	78.80±1.81^a^	1.73±0.04^a^	n.d.	n.d.	0.62±0.47^a^	9.35±0.24^a^	n.d.	n.d.	n.d.	0.69±0.02^a^	11.66±0.29^a^

Values are presented as mean ± standard deviation; Del-3-Glc-Delphinidin-3-glucoside, Cya-3-Glc-Cyanidin-3-glucoside, Pet-3-Glc-Petunidin-3-glucoside, Peo-3-Glc-Peonidin-3-glucoside, Mal-3-Glc-Malvidin-3-glucoside, Del-3-AcGlc-Delphinidin-3-acetylglucoside, Cya-3-AcGlc-Cyanidin-3-acetylglucoside, Pet-3-AcGlc-Petunidin-3-acetylglucoside, Peo-3-AcGlc-Peonidin-3-acetylglucoside, Mal-3-AcGlc-Malvidin-3-acetylglucoside, Del-3-CoGlc-Delphidin-3-coumaryl-glucoside, Cya-3-CoGlc-Cyanidin-3-coumaroylglucoside, Pet-3-CoGlc-Petunidin-3-coumaroylglucoside, Peo-3-CoGlc-Peonidin-3-coumaroylglucoside; Mal-3-CoGlc-Malvidin-3-coumaroylglucoside. Means within a column followed by the same letter are not significantly different ANOVA and Tukey post-hoc test (*p*˂0.05). Spiked red wine (TF) and wine treated with chitins (CHTN, CHTNA at 10 g/h L) and chitosans (CHTB, CHTC, CHTD, CHTF at 10 g/h L).

**Table 8:** Total phenols, flavonoid phenols, non-flavonoid phenols, total anthocyanins and chromatic characteristics of red wines before (TF) and after treatment with chitosans with different physicochemical characteristics and application doses.

**Samples**	**Total phenols**	**Flavonoid phenols**	**Non-flavonoid phenols**	**Total anthocyanins**	**Colour intensity**	**Hue**	L*	a*	b*	C*	**°*h***	**Δ*E***
	**(mg/L gallic acid)**	**(mg/L gallic acid)**	**(mg/L gallic acid)**	**(mg/L)**	**A.U.**							
**TF**	1921±6^c^	1538±8^a^	383±2^ab^	364±2^ab^	11.06±0.99^ab^	0.60±0.09^b^	10.88±1.08^abc^	41.19±2.19^abc^	38.86±1.53^ab^	56.63±2.64^ab^	0.76±0.01^a^	…
**CHTD10**	1877±20^bc^	1466±12^ab^	411±8^b^	371±11^a^	12.29±0.96^b^	0.70±0.01^ab^	8.52±0.95^b^	37.38±1.05^c^	35.54±1.25^a^	51.58±2.17^b^	0.76±0.01^a^	5.24±1.83^a^
**CHTD100**	1745±37^ab^	1353±23^bcd^	392±14^ab^	361±10^ab^	11.43±0.20^ab^	0.71±0.01^ab^	8.02±0.86^bc^	36.56±1.87^ac^	34.38±1.67^ab^	50.19±2.51^ab^	0.75±0.00^a^	6.74±0.23^ab^
**CHTD500**	1567±15^d^	1225±19^d^	342±5^c^	325±2^c^	7.94±0.05^cd^	0.77±0.00^a^	15.06±0.02^a^	44.99±0.05^ab^	35.13±0.24^ab^	57.08±0.11^ab^	0.66±0.00^d^	7.69±0.01^ab^
**CHTF10**	1845±37^abc^	1445±44^abc^	400±7^ab^	373±4^a^	10.84±0.49^ab^	0.66±0.00^ab^	12.18±0.92^abc^	42.88±1.81^abc^	39.84±1.02^b^	58.54±2.02^a^	0.75±0.01^ab^	2.35±0.60^b^
**CHTF100**	1719±77^a^	1316±74^cd^	403±2^ab^	364±10^ab^	9.83±0.33^ad^	0.67±0.01^ab^	13.26±0.49^ac^	43.94±0.84^ab^	38.93±0.10^ab^	58.71±0.70^a^	0.73±0.01^b^	3.63±1.53^ab^
**CHTF500**	1431±21^d^	1051±23^e^	380±2^a^	339±3^bc^	7.24±0.35^c^	0.78±0.00^a^	16.80±1.32^a^	45.91±1.90^b^	33.53±0.73^a^	56.85±1.97^ab^	0.63±0.01^c^	9.26±2.75^a^

Values are presented as mean ± standard deviation; Means within a column followed by the same letter are not significantly different (Tukey, *p*˂0.05). L* – lightness, a* - redness, b* - yellowness, ΔE* – colour difference. The values corresponding to ΔE* were obtained taking as a reference the untreated wine (TF). A.U. – absorbance units, spiked red wines (TF) and wines treated with chitosans (CHTD and CHTF at 10, 100 and 500 g/h L).

**Table 9:** Phenolic acids and flavonoids of red wines spiked with volatile phenols (TF) and after treatment with chitosans with different physicochemical characteristics and application doses.

**Samples**	**Gallic acid**	**Catechin**	***trans*****-caftaric acid**	**GRP**	**Coutaric acid**	**Caffeic acid**	***p*****-Coumaric acid**	**Ferulic acid**	**Caffeic acid ethyl ester**	***p*-Coumaric acid ethyl ester**
	**(mg/L)**	**(mg/L)**	**(mg/L)**	**(mg/L)**	**(mg/L)**	**(mg/L)**	**(mg/L)**	**(mg/L)**	**(mg/L)**	**(mg/L)**
**TF**	42.61±0.14^a^	26.13±0.71^a^	21.67±0.09^a^	n.d.	12.23±0.07^e^	6.87±0.01^a^	1.98±0.13^a^	2.55±0.07^a^	1.68±0.01^ab^	3.39±0.08^ab^
**CHTD10**	41.77±0.30^a^	26.48±0.76^a^	20.19±0.17^a^	n.d.	10.97±0.09^cd^	6.04±0.11^a^	2.02±0.03^a^	2.51±0.06^a^	1.32±0.12^a^	3.01±0.05^a^
**CHTD100**	41.52±0.69^a^	25.73±1.33^ab^	16.40±0.54^c^	n.d.	9.68±0.18^b^	5.91±0.17^a^	2.08±0.06^a^	2.43±0.03^a^	1.56±0.00^ab^	3.11±0.22^ab^
**CHTD500**	34.37±0.96^b^	24.03±0.06^ab^	9.54±0.39^b^	n.d.	6.09±0.03^a^	5.64±0.04^a^	1.84±0.01^a^	2.37±0.08^ab^	1.64±0.01^ab^	3.06±0.11^a^
**CHTF10**	42.20±0.02^a^	26.46±0.90^a^	21.25±0.20^a^	n.d.	11.86±0.10^de^	7.00±0.04^a^	2.26±0.12^a^	2.58±0.06^a^	1.92±0.18^b^	4.17±0.45^b^
**CHTF100**	41.33±0.96^a^	26.17±0.86^a^	18.12±0.72^d^	n.d.	10.52±0.67^bc^	6.75±0.25^a^	2.16±0.20^a^	2.54±0.02^a^	1.64±0.25^ab^	3.35±0.49^ab^
**CHTF500**	35.81±1.07^b^	22.77±0.73^b^	8.13±0.42^b^	n.d.	5.73±0.18^a^	6.51±0.40^a^	2.11±0.12^a^	2.17±0.06^b^	1.69±0.03^ab^	3.07±0.08^a^

Values are presented as mean ± standard deviation; Means within a column followed by the same letter are not significantly different (Tukey, *p*<0.05).

GRP - 2-*S*-glutathionyl caftaric acid. Spiked red wine (TF) and wine treated with chitosans (CHTD and CHTF at 10, 100 and 500 g/h L).

**Table 10:** Monomeric anthocyanin composition of spiked red wines (TF) and after treatment with chitosans with different physicochemical characteristics and application doses.

**Samples**	**Del-3-Glc**	**Cya-3-Glc**	**Pet-3-Glc**	**Peo-3-Glc**	**Mal-3-Glc**	**Del-3-AcGlc**	**Cya-3-AcGlc**	**Pet-3-AcGlc**	**Peo-3-AcGlc**	**Mal-3-AcGlc**	**Del-3-CoGlc**	**Cya-3-CoGlc**	**Pet-3-CoGlc**	**Peo-3-CoGlc**	**Mal-3-CoGlc**
	**(mg/L)**	**(mg/L)**	**(mg/L)**	**(mg/L)**	**(mg/L)**	**(mg/L)**	**(mg/L)**	**(mg/L)**	**(mg/L)**	**(mg/L)**	**(mg/L)**	**(mg/L)**	**(mg/L)**	**(mg/L)**	**(mg/L)**
**TF**	0.53 ±0.06^a^	7.26±0.74^a^	9.20±0.89^a^	4.19±0.44^a^	66.78±0.82^a^	0.94±0.23^a^	n.d.	n.d.	0.40±0.00^a^	7.82±1.22^a^	n.d.	n.d.	n.d.	0.47±0.07^a^	5.14±0.59^a^
**CHTD10**	0.51±0.04^a^	5.50±0.23^b^	7.18±0.10^a^	3.37±0.02^a^	67.70±1.30^a^	0.78±0.05^a^	n.d.	n.d.	n.d.	6.62±0.05^a^	n.d.	n.d.	n.d.	0.43±0.01^a^	4.31±0.16^a^
**CHTD100**	0.51±0.02^a^	5.48±0.19^ab^	8.08±0.24^a^	3.32±0.01^a^	65.89±1.68^a^	0.76±0.05^a^	n.d.	n.d.	n.d.	6.60±0.09^a^	n.d.	n.d.	n.d.	0.42±0.00^a^	4.34±0.30^a^
**CHTD500**	0.58±0.04^a^	5.41±0.23^ab^	7.70±0.26^a^	3.34±0.56^a^	66.35±0.93^a^	0.77±0.16^a^	n.d.	n.d.	n.d.	6.54±0.69^a^	n.d.	n.d.	n.d.	n.d.	4.03±0.23^a^
**CHTF10**	0.57±0.01^a^	7.57±0.45^a^	9.66±0.75^a^	4.14±0.61^a^	71.93±0.50^a^	0.93±0.18^a^	n.d.	n.d.	0.38±0.04^a^	8.16±1.36^a^	n.d.	n.d.	n.d.	0.59±0.05^a^	5.63±0.31^a^
**CHTF100**	0.57±0.07^a^	6.79±0.86^a^	8.70±1.21^a^	3.76±0.32^a^	68.77±0.80^a^	0.93±0.19^a^	n.d.	n.d.	0.36±0.03^a^	8.22±0.45^a^	n.d.	n.d.	n.d.	0.50±0.00^a^	4.72±0.77^a^
**CHTF500**	0.56±0.02^a^	5.96±0.17^a^	8.08±0.05^a^	3.89±0.03^a^	68.89±0.88^a^	0.86±0.26^a^	n.d.	n.d.	n.d.	7.12±0.28^a^	n.d.	n.d.	n.d.	0.50±0.10^a^	4.26±0.08^a^

Values are presented as mean ± standard deviation; Del-3-Glc-Delphinidin-3-glucoside, Cya-3-Glc-Cyanidin-3-glucoside, Pet-3-Glc-Petunidin-3-glucoside, Peo-3-Glc-Peonidin-3-glucoside, Mal-3-Glc-Malvidin-3-glucoside, Del-3-AcGlc-Delphinidin-3-acetylglucoside, Cya-3-AcGlc-Cyanidin-3-acetylglucoside, Pet-3-AcGlc-Petunidin-3-acetylglucoside, Peo-3-AcGlc-Peonidin-3-acetylglucoside, Mal-3-AcGlc-Malvidin-3-acetylglucoside, Del-3-CoGlc-Delphidin-3-coumaryl-glucoside, Cya-3-CoGlc-Cyanidin-3-coumarylglucoside, Pet-3-CoGlc-Petunidin-3-coumarylglucoside, Peo-3-CoGlc-Peonidin-3-coumarylglucoside; Mal-3-CoGlc-Malvidin-3-coumarylglucoside. Means within a column followed by the same letter are not significantly different ANOVA and Tukey post-hoc test (*p*<0.05). Spiked red wine (TF) and wine treated with chitosans (CHTD and CHTF at 10, 100 and 500 g/h L).

## 2 Experimental design, materials and methods

### 2.1 Chitin and chitosan samples and production

Commercial crustacean chitin (CHTN, Chitin from shrimp shells, Sigma C9213), two commercial crustacean chitosans (CHTB, Chitosan high molecular weight, Sigma 419419 and CHTD, Chitosan 100000–300000 Da, Acros 34905500) and one fungal chitosan (CHTF, No Brett Inside, Lallemand) where used. One additional chitin (CHTNA) and one additional chitosan (CHTC) were produced by alkaline deacetylation of CHTN and CHTB, respectively [1]. For deacetylation of chitin and chitosan, 15 g of the initial material were dispersed in 150 mL NaOH solution (50% w/v) with NaBH_4_ (10 g/L) and heated during 12 h under reflux with stirring, at 130–150 °C under nitrogen [10]. For chitin deacetylation, commercial chitin was previously grounded to particles size less than 0.15 mm (obtained by sieving). After cooling to room temperature, the solution was neutralised to pH 6–8 with HCl 12 M and ethanol was added until 75% (v/v) for chitosan precipitation. The precipitate was washed thoroughly with ethanol at 75% (v/v). The material was dried at 50 °C in a forced air oven during 24 h.

### 2.2 Chitin and chitosan chemical characterisation

#### 2.2.1 Chitin and chitosan degree of deacetylation

Chitin and chitosan DD were determined by potentiometric titration [11]. To 200 mg of chitosan, 50 mL of 0.02 mol/L HCl were added and the dispersion was stirred at room temperature during 24 h for obtaining maximum or total solubilisation. The final solution was titrated with previously standardised 0.01 mol/L NaOH and the first and second end-points were determined by potentiometrically using a pH glass electrode. The DD was determined using the following equation (Eq. (1)):

(1) $$\%\mathrm{DD}=\frac{161\times C_{\mathrm{NaOH}}(v_{2}-v_{1})}{m}\times 100$$

where %DD is the percentage of deacetylation degree, *v*_*1*_ is the volume, in mL, of NaOH used to neutralize the excess of HCl in solution, *v*_*2*_ is the volume, in mL, of NaOH used to neutralise the amine groups in chitosan, *161* corresponds to the molecular weight of anhydroglucosamine and *m* is the quantity, in mg, of chitosan. Analyses were performed in triplicate.

#### 2.2.2 Viscosity average molecular weight of chitosans

The molecular weight and viscosity behaviour of chitosan was determined using Ubbelohde capillary viscometer (N° 0B, ASTM-D2515) at 25 °C, having a flow time for the solvent used of 195 seconds (*t*_0_). Chitosan solutions of different concentrations (0.1 to 1 g/L or 0.4 g/L to 4.0 g/L) in 2% acetic acid, 0.2 mol/L sodium acetate (pH 4.5) solutions were prepared [12]. All the solutions were magnetically stirred for 1 hour in order to ensure proper dissolution of chitosan. The flow times of chitosan solutions and solvent were recorded in triplicate and the average value was calculated. The intrinsic viscosity [η] was calculated graphically by extrapolating the curve of specific viscosity (Eq. (2)) and reduced viscosity (Eq. (3)) versus concentration to zero concentration.

(2) $$\eta_{\mathrm{sp}}=\frac{t-t_{0}}{t_{0}}$$

(3) $$\eta_{\mathrm{red}}=\frac{\eta_{\mathrm{sp}}}{C}$$

where *t*_*0*_ is the solvent flow time in seconds, *t* is the flow time of the chitosan solutions in seconds and *C* is the concentration of the chitosan solution in g/L. The molecular weight of chitosan was obtained according to the Mark-Houwink equation (Eq. (4)) [12]:

(4) $$\left[\eta \right]=KM_{v}^{a}$$

where [η] in L/g is the intrinsic viscosity of the polymer, *M*_*v*_ is the viscosity average molecular weight of the polymer and *K* and *a* are the characteristic constants of the polymer-solvent system (*K*=1.38×10^−5^; *a*=0.85) [13].

#### 2.2.3 FTIR analysis of chitins and chitosans

Chitin and chitosan FTIR spectra were recorded in the range of wavenumbers 4000–450 cm^−1^ and 128 scans were taken at 2 cm^−1^ resolution, using a Unicam Research Series FTIR spectrometer. Pellets were prepared by thoroughly mixing samples with KBr at a 1:40 sample/KBr weight ratio in a small size agate mortar. The resulting mixture was placed in a manual hydraulic press, and a force of 10 t was applied for 10 min. The spectra obtained were background corrected and smoothed using the Savitzky-Golay algorithm using PeakFit v4 (AISN Software Inc., 1995). Analyses were performed in duplicate.

#### 2.2.4 X-Ray diffraction analysis of chitins and chitosans

Powder X-ray diffraction (XRD) data were recorded on solid samples (chitins and chitosans) using a PANalytical X’Pert Pro X-ray diffractometer equipped with X’Celerator detector and secondary monochromator. The measurements were carried out using a Cu Kα radiation (40 kV; 30 mA) in Bragg-Bentano geometry at 7–60° 2θ angular range. Analyses were performed in duplicate.

### 2.3 Experimental design

For studying the effect of DD on chitins and chitosans headspace volatile phenols reduction performance, two chitins and four different chitosans were used at 10 g/h L (CHTN10, CHTNA10, CHTB10, CHTC10, CHTD and CHTF10). The wine was previously spiked at two levels of 4-EP (750 and 1500 µg/L) and 4-EG (150 and 300 µg/L), named 4-EP750, 4-EP1500, and 4-EG150, 4-EG300, respectively, according to the ranges usually found in the literature [14], [15], [16]. Chitins and chitosans were added at 10 g/h L, to the wine placed in 250 mL graduated cylinders. For studying the effect of chitosan application dose, the chitosans CHTD and CHTF were also tested in a second trial at 10, 100 and 500 g/h L (CHTD10, CHTD100, CHTD500, CHTF10, CHTF100 and CHTF500). After 6 days the wine was centrifuged at 10,956*g*, 10 min at 20 °C for analysis. All experiments were performed in duplicate.

### 2.4 Analysis of conventional oenological parameters

The analysis of conventional oenological parameters (alcohol content, specific gravity, pH, titratable and volatile acidity) were analysed using a FTIR Bacchus Micro (Microderm, France).

### 2.5 Wine samples

In this work were used two blend red wines from Douro Valley (vintage 2015). Wine main characteristics used in the first assay (CHTN10, CHTNA10, CHTB10, CTHC10, CHTD10, CHTF10), were as follows: alcohol content 13.3% (v/v), specific gravity (20 °C) 0.9921 g/mL, titratable acidity 5.7 g of tartaric acid/L, pH 3.52, volatile acidity 0.54 g of acetic acid/L, total phenolic compounds 1907 mg of gallic acid equivalents/L, total anthocyanins 343 mg of malvidin-3-glucoside equivalents/L. In the second assay the wine used (CHTD10, CHTD100, CHTD500, CHTF10, CHTF100, CHTF500) presented an alcohol content of 13.4% (v/v), specific gravity (20 °C) 0.9935 g/mL, titratable acidity 5.5 g of tartaric acid/L, pH 3.56, volatile acidity 0.43 g of acetic acid/L, total phenolic compounds 1921 mg of gallic acid equivalents/L, total anthocyanins 364 mg of malvidin-3-glucoside equivalents/L.

### 2.6 Headspace wine aroma abundance by solid phase microextraction (HS-SPME)

For the determination of the headspace aroma abundance of red wines a validated method, confirmed in our laboratory was used [4]. Briefly the fibre used was coated with Divinylbenzene/Carboxen/Polydimethylsiloxane 50/30 μm (DVB/CAR/PDMS) and was conditioned before use by insertion into the GC injector at 270 °C for 60 min (Trace GC, Polaris Q MS, Thermo). To a 20 mL headspace vial, 10 mL of wine, 2.5 g/L of NaCl and 50 µL (500 mg/L) of 3-octanol us an internal standard was added. The vial was sealed with a Teflon septum. The fibre was inserted through the vial septum and exposed during 60 min to perform the extraction by an automatic CombiPal system at 35 °C. The fibre was inserted into the injection port of the GC during 3 min at 270 °C. For separation an Optima-FFAP column (30 m×0.32 mm ID, Macherey-Nigel, Germany) was used. The temperature program was as follows: initial temperature 40 °C hold during 2 min, followed by an increase in temperature at 2 °C/min to 220 °C followed by an increase at 10 °C/min to 250 °C, hold during 3 min. The flow rate was set at 1.5 mL/min and maintained constant during the run. The transfer line temperature was 250 °C and the ion source was set at 220 °C. The mass scan was performed between *m/z* 45 and 650, the scan event was 0.59 s. All analyses were performed in quadruplicate.

### 2.7 Analysis of wine glucosamine content

In the second assay, for quantification of the wine glucosamine content, wines treated with 10, 100 and 500 g/h L of chitosans CHTD and CHTF were performed as follows: to 4 mL of wine, 400 μL of 72% H_2_SO_4_, and the samples were heated at 100 °C for 2.5 h. After hydrolysis, 500 μL of 2-deoxyglucose at 1 mg/mL was added as an internal standard and the glucosamine content was determined by anion-exchange chromatography using the method described by Ribeiro et al. [17]. Under the conditions of the analytical method the lowest standard in the calibration curve (presenting a signal to noise ratio higher than 10) corresponds to 4.5 mg of anhydrous glucosamine/L of wine. Analyses were performed in quadruplicate.

### 2.8 Colour, total anthocyanins and chromatic characterisation

Colour intensity and hue were determined according to OIV [18]. The content of total anthocyanins was determined according to Ribéreau-Gayon and Stonestreet [19]. Wine chromatic characterisation [L*(lightness), a* (redness), and b* (yellowness) coordinates] were calculated using the CIELab method according to OIV [18]. The Chroma [C*=[(a*)^2^+(b*)^2^]^1/2^] and hue-angle [h°=tang^_1^ (b*/a*)] values were also determined. To distinguish the colour more accurately, the colour difference was calculated using the following equation: ΔE*=[(ΔL*)^2^+(Δa*)^2^+(Δb*)^2^]^1/2^. All analyses were performed in duplicate.

### 2.9 Quantification of non-flavonoids, flavonoids and total phenols

The wine non-flavonoids content was quantified according to Kramling and Singleton [20]. The results were expressed as gallic acid equivalents by means of calibration curves with standard gallic acid. The total phenolic content was determined according to Ribéreau-Gayon et al. [21]. All analyses were performed in duplicate.

### 2.10 High performance liquid chromatography (HPLC) analysis of anthocyanins and phenolic acids

Analyses were performed with an Ultimate 3000 HPLC equipped with a PDA-100 photodiode array detector and an Ultimate 3000 pump. The separation was performed on a C18 column (250 mm×4.6 mm, 5 μm particle size) with a flow rate of 1 mL/min at 35 °C. The injection volume was 50 μL and the detection was performed from 200 to 650 nm with 75 min per sample. The analyses conditions were carried out using 5% aqueous formic acid (A) and methanol (B) and the gradient was as follows: 5% B from zero to 5 min followed by a linear gradient up to 65% B until 65 min and from 65 to 67 min down to 5% B [22]. Quantification was carried out with calibration curves with standards caffeic acid, coumaric acid, ferulic acid, gallic acid and catechin. The results of *trans*-caftaric acid, 2-*S*-glutathionylcaftaric acid (GRP) and caffeic acid ethyl ester were expressed as caffeic acid equivalents by means of calibration curves with standard caffeic acid. On the other hand, coutaric acid, coutaric acid isomer and *p*-coumaric acid ethyl ester were expressed as coumaric acid equivalents by means of calibration curves with standard coumaric acid. A calibration curve of cyanidin-3-glucoside (y (Area)=2.70×(mg/L)+0.00; r=0.99980), malvidin-3-glucoside (y (Area)=1.62×(mg/L)+0.14; r=0.99985), peonidin-3-glucoside (y (Area)=2.49×(mg/L)+0.19; r=0.99994) and pelargonidin-3-glucoside (y (Area)=1.66×(mg/L)+0.99; r=0.99990) was used for quantification of anthocyanins. Using the coefficient of molar absorptivity (ε) and by extrapolation, it was possible to obtain the slopes for delphinidin-3-glucoside (ε=23700 L mol^−1^ cm^−1^), petunidin-3-glucoside (ε=18900 L mol^−1^ cm^−1^) and malvidin-3-coumaroylglucoside (ε=20200 L mol^−1^ cm^−1^) to perform the quantification [23]. The results of delphinidin-3-acetylglucoside, petunidin-3-acetylglucoside, peonidin-3-acetylglucoside, cyanidin-3-acetylglucoside and cyanidin-3-coumarylglucoside were expressed as respective glucoside equivalents.

### 2.11 Statistical treatment

The data are presented as means ± standard deviation. To determine whether there is a statistically significant difference between the data obtained for the diverse parameters quantified in the red wines, an analysis of variance (ANOVA, one-way) and comparison of treatment means were carried out. Tukey honestly significant difference (HSD, 5% level) test was applied to physicochemical data to determine significant differences between treatments. All analyse were performed using Statistica 10 Software (StatSoft, Tulsa, OK U.S.A.).
