# Supplementary material for: Data on changes in red wine phenolic compounds and headspace aroma compounds after treatment of red wines with chitosans with different structures
Source: Data Brief. 2018 Feb 16;17:1201–17. doi: 10.1016/j.dib.2018.02.029 (PMC5988412; doi:10.1016/j.dib.2018.02.029)
Supplement: Supplementary file 1 — Supplementary material [file mmc1.docx]

We declare no conflict of interest
